# Initial Insights Into an Institutional Secure Large Language Model for Magnetic Resonance Imaging Examination Requests: Retrospective Study

**DOI:** 10.2196/82579

**Published:** 2026-04-07

**Authors:** James Thomas Patrick Decourcy Hallinan, Naomi Wenxin Leow, Yi Xian Low, Aric Lee, Wilson Ong, Matthew Ding Zhou Chan, Ganakirthana Kalpenya Devi, Stephanie Shengjie He, Daniel De-Liang Loh, Desmond Shi Wei Lim, Xi Zhen Low, Mei Chin Lim, Clement Yong, Weizhong Jonathan Sng, Ee Chin Teo, Jiong Hao Tan, Naresh Kumar, Andrew Makmur, Yonghan Ting

**Affiliations:** 1 Department of Diagnostic Imaging, National University Hospital, 5 Lower Kent Ridge Rd, Singapore, 119074, Singapore, 65 6908 2222; 2 Department of Diagnostic Radiology, Yong Loo Lin School of Medicine, National University of Singapore, Singapore, Singapore; 3 Innovation Office, AI Office, National University Health System, Singapore, Singapore; 4 National University Spine Institute, Department of Orthopedic Surgery, National University Hospital, Singapore, Singapore

**Keywords:** secure large language model, radiology request form, reason for exam imaging reporting and data system, musculoskeletal imaging, body imaging, neuroradiology imaging, magnetic resonance imaging

## Abstract

**Background:**

Incomplete clinical details on magnetic resonance imaging (MRI) examination requests (MERs) can lead to suboptimal protocol selection. An institutional secure large language model (sLLM) with access to manually retrieved salient data from the electronic medical record (EMR) may improve request completeness and protocol accuracy across multiple MRI subspecialties.

**Objective:**

The objective of this study was to compare clinician MERs with sLLM-augmented MERs for information quality and to evaluate the protocoling accuracy of the sLLM versus board-certified radiologists across body, musculoskeletal, and neuroradiology MRI.

**Methods:**

This retrospective study included 608 random outpatient MRI examinations performed between September 2023 and July 2024 (body 206, musculoskeletal 203, neuroradiology 199). The cohort comprised 528 patients (mean 51.2 years, SD 19.2; range 4‐93; n=279, 52.8% women, n=249, 47.2% men). MERs without EMR access were excluded. A privately hosted Anthropic Claude 3.5 model (temperature 0) augmented each MER with manually retrieved salient EMR data and, via rule-based parsing, mapped the extracted elements onto predefined institutional criteria to recommend region or coverage and contrast use. Two experienced radiologists established a consensus reference standard. Two board-certified general radiologists (Rad 3 and Rad 4) and the sLLM were compared with this standard. Clinical information quality was graded using the Reason-for-Exam Imaging Reporting and Data System (RI-RADS). Interrater reliability was quantified with Gwet AC1. Paired accuracies were compared with the McNemar test to determine whether there was a statistically significant difference.

**Results:**

Interreader agreement for RI-RADS was almost perfect for sLLM-augmented MERs (AC1 0.97, 95% CI 0.94‐0.99) and moderate for clinician MERs (AC1 0.43, 95% CI 0.34‐0.52). Limited or deficient clinical information (RI-RADS C/D) fell to 0% to 0.7% (0/608 to 4/608) with sLLM augmentation vs 4.1% to 20.4% (25/608 to 124/608) for clinician MERs. Overall protocol accuracy was 93.1% (566/608; 95% CI 89.6‐96.6) for the sLLM, 91.4% (556/608; 95% CI 87.6‐95.3) for Rad 3, and 92.1% (560/608; 95% CI 88.4‐95.8) for Rad 4 (sLLM vs Rad 3 *P*=.23 vs Rad 4 *P*=.40). Region or coverage accuracy was similar (sLLM: 579/608, 95.2%; Rad 3: 585/608, 96.2%; Rad 4: 573/608, 94.2%; *P*=.46 and *P*=.36). Contrast decisions were more accurate using the sLLM at 94.4% (574/608; 95% CI 91.3‐97.5) vs Rad 3 at 92.1% (560/608; 95% CI 88.4‐95.8; *P*=.027) and were not significantly different to Rad 4 at 92.9% (565/608; 95% CI 89.4‐96.4; *P*=.16). Subspecialty analyses showed similar patterns, with the sLLM outperforming Rad 4 for musculoskeletal MRI contrast decisions (96.6% vs 91.1%; *P*=.006) and matching readers elsewhere. Manual review indicated that sLLM improvements arose from EMR details not listed on the MER (infection/inflammation, tumor history, prior surgery). No clinically significant hallucinations were identified in a manual review of discordant cases.

**Conclusions:**

Across body, musculoskeletal, and neuroradiology MRI, sLLM-augmented examination requests improved clinical context and enhanced contrast selection while demonstrating accuracy comparable to general radiologists for region or coverage. Integrating sLLMs into routine vetting workflows may reduce manual workload in protocol selection for more efficient, standardized protocoling.

## Introduction

Radiology examination requests form the primary communication bridge between referring clinicians and radiology services. Completeness and clarity of these requests influence protocol selection, image quality, and ultimately diagnostic and therapeutic outcomes [1-3]. When key clinical elements such as relevant history, current symptoms, or prior imaging are incomplete or absent, radiographers and radiologists may assign a suboptimal protocol or omit intravenous contrast, leading to lower accuracy, repeat studies, and additional cost [4-6]. A recent systematic review showed that incomplete clinical information was associated with reduced reporting accuracy, clinical relevance, and reporting confidence [5].

The enhancement of radiology examination requests with data from the electronic medical record (EMR) offers a potential solution. Early rule-based decision-support tools reduced certain categories of inappropriate imaging yet may struggle to accommodate unstructured narratives and updated imaging protocols [6]. Natural language processing pipelines improved specificity but required extensive engineering and did not fully interpret clinical context [7]. Current large language models (LLMs) can ingest heterogeneous text, interpret medical terminology, and generate structured outputs at scale [8-13]. Proof-of-concept work has already demonstrated utility for report generation and national guideline concordance [14,15]. Nevertheless, many published studies rely on deidentified vignettes or synthetic notes, limiting direct clinical translation because of privacy constraints.

Secure LLMs (sLLMs) address these constraints by operating behind institutional firewalls while preventing the transmission of protected health information [16]. Several studies have demonstrated their feasibility, including improving the adequacy of spine magnetic resonance imaging (MRI) request forms and increased protocol concordance for musculoskeletal studies [17,18]. Recent oncologic imaging work further indicates that a GPT-4 system can automatically generate structured clinical histories that radiologists prefer over original clinician-generated requisitions [19]. Another recent study on GPT-4-generated MRI protocols showed notable quality in cardiac and neuroradiology imaging [20]. An accompanying editorial emphasized that fatigue-free, verifiable LLM summaries could finally bridge the long-recognized information gap between referrers and radiologists, reinforcing the case for secure, institution-hosted models [21].

Building on these findings, we evaluated a secure LLM across all routine MRI subspecialties, including body, neuroradiology, and musculoskeletal examinations. The study had 2 objectives: (1) to compare the information quality of clinician MRI examination requests (MERs) with those augmented by the sLLM using the Reason-for-Exam Imaging Reporting and Data System (RI-RADS) [22] and (2) to assess protocol accuracy of the sLLM against both subspecialty and general radiologist readers.

## Methods

### Ethical Considerations

The Institutional Review Board (Domain-Specific Review Board reference: 2023/00919) classified the project as minimal risk and therefore granted a waiver of informed consent. Patient-identifying details have been omitted to maintain the privacy and confidentiality of patient data. Compensation was not required in view of the minimal risk of the project.

### Protocoling Pipeline and sLLM Prompt

Original clinician-submitted outpatient MERs performed between September 2023 and July 2024 were retrieved at random. MERs lacking corresponding EMR information were excluded. The musculoskeletal cohort in this study represents a distinct nonoverlapping dataset from that of a previous study by the authors. A target sample of approximately 600 requests (~200 per major subspecialty) was selected to provide adequate statistical power while maintaining operational feasibility across a diverse range of anatomical regions and pathologies. For every patient, a MER was generated by the sLLM based on the clinician request with relevant EMR content.

The institutional sLLM is a privately hosted instance of Anthropic Claude 3.5 on Amazon Bedrock. Model temperature was fixed at 0 to minimize output variability. For each request, the sLLM received (1) the most recent relevant clinical entry identified manually by the authors, based on the clinical service indicated on the request forms and (2) pertinent prior imaging reports (eg, CT neck for an MRI nasopharynx study). The typical input length of the clinical notes provided to the sLLM ranged from around 100 words for routine or straightforward presentations to around 3000 words for patients with complex oncological or multisystem disease. The sLLM was also given the latest institutional MRI protocol repository (Multimedia Appendix 1). No clinical data left the institutional firewall.

Two-step sLLM protocoling was performed:

1. Information enrichment—the sLLM extracted key findings, working diagnoses, and potential MRI safety issues from both the MER and the EMR, returning a concise justification summary.
2. Protocol assignment—using institutional rules and the enriched summary, the model selected the optimal region/coverage and determined whether contrast was required.

The estimated processing cost using the sLLM was US $3 per 1,000,000 tokens for the input and US $15 per 1,000,000 tokens for the output, giving a total cost of US $0.024 to 0.033 per request. For the cases requiring longer input length (about 5000 tokens), the per-case cost remained low at a few cents per request.

Importantly, the sLLM’s primary role in this pipeline was clinical information extraction, summarizing clinically relevant details from the MER and EMR (eg, prior surgery, suspected infection, etc) and contextual interpretation. The final contrast and region decisions were not made autonomously by the sLLM. Instead, the extracted elements were subsequently mapped onto predefined institutional criteria for region or coverage and contrast use using a deterministic rule-based parsing script.

Contrast (gadolinium) administration was considered when any of the following were present:

1. History or suspicion of tumor, malignancy, or focal lesion
2. Known or suspected infective or inflammatory condition
3. Injury to neural structures
4. Previous surgery or spinal/extremity instrumentation
5. Explicit clinician request

A custom parsing script evaluated the sLLM response, which listed a “yes” or “no” decision for each contrast-relevant category together with the reason when applicable. If the script detected at least one positive flag and renal failure or other contraindications had not been recognized, the examination was classified as “contrast required.”

Detailed prompt wording and rule logic are provided in Multimedia Appendix 1. The rule definitions in this study were refined through prior work on LLMs in spine and musculoskeletal protocoling. All existing MRI protocols (including prompt design and contrast rules) were carefully reviewed with subspecialty leads and key team members to ensure alignment with institutional practice before doing the formal analysis. The full institutional MRI protocol table had a size compatible with the available context window and was embedded directly within the prompt, obviating the need for retrieval-augmented generation (RAG) in this study. No cases from the present study were used during prompt development.

### Evaluation Procedure

Two senior radiologists (Rad 1: 14 years of experience; Rad 2: 12 years of experience) independently graded the clinical adequacy of both clinician and sLLM MERs using the RI-RADS classification (Multimedia Appendix 2). They were blinded to the origin of each form.

Rad 1 and Rad 2 provided a consensus reference standard for protocoling. Against this consensus reference standard, protocol selections generated by the sLLM were then compared with those from 2 board-certified general radiologists (Rad 3 and Rad 4), who also had access to the same EMR entries, pertinent prior imaging reports, and institutional MRI protocols. The pertinent prior imaging reports were manually selected by study members, who were individuals distinct from Rad 1 and Rad 2. Both Rad 3 and Rad 4 had 2 years of experience and were commonly tasked with providing protocols based on MRI examination requests. Accuracy was credited only when the suggested region or coverage and contrast decision matched the consensus reference standard. Additional subanalyses were performed for each component and the major subspecialties. A manual review of disagreements between the sLLM and board-certified general radiologists against the consensus reference standard was carried out by the senior radiologists. Note was made of any hallucinations by the sLLM with clinically significant hallucinations defined as the sLLM producing clinically relevant details absent from the provided input data and capable of influencing protocol decisions [23].

### Statistical Analysis

All computations were performed with Python 3.9.12. Two-sided tests were considered significant at *P*<.05.

The quality of each MER was rated on a 4-level scale (RI-RADS grades A-D) by Rad 1 and Rad 2. Interreader agreement for these ordinal ratings, and later for MRI protocoling decisions (region/coverage and contrast requirement), was quantified with Gwet AC1, which is less sensitive than Cohen κ to category imbalance (eg, the predominance of A/B grades) [24]. Agreement was interpreted as poor (<0), slight (0‐0.20), fair (0.21‐0.40), moderate (0.41‐0.60), substantial (0.61‐0.80), or almost perfect (0.81‐1). With more than 600 examinations, the study had greater than 90% power to detect a minimum 15% absolute difference in the proportion of clinically adequate MERs between clinician and sLLM versions.

Protocol selections generated by the sLLM and by 2 board-certified general radiologists (Rad 3 and Rad 4) were compared with the consensus reference standard established by Rad 1 and Rad 2. Overall accuracy was defined as the proportion of cases in which both region or coverage and contrast indication matched the reference standard. Differences in paired accuracies (sLLM vs each radiologist) were evaluated using the McNemar test with Yates continuity correction. Separate analyses were undertaken for region alone and contrast alone, and across the 3 major subspecialties (body, musculoskeletal, and neuroradiology). Ninety-five percent CIs for RI-RADS gradings (quality of clinical information) and protocoling were derived from the normal approximation to the binomial distribution. For proportions near 0% or 100%, the normal approximation to the binomial can be inaccurate. Therefore, for low-event RI-RADS C/D proportions (eg, sLLM MERs), 95% CIs were computed using the Clopper-Pearson exact method rather than the normal approximation.

## Results

### Patient Demographics and Consensus MRI Protocols

**Figure 1.**
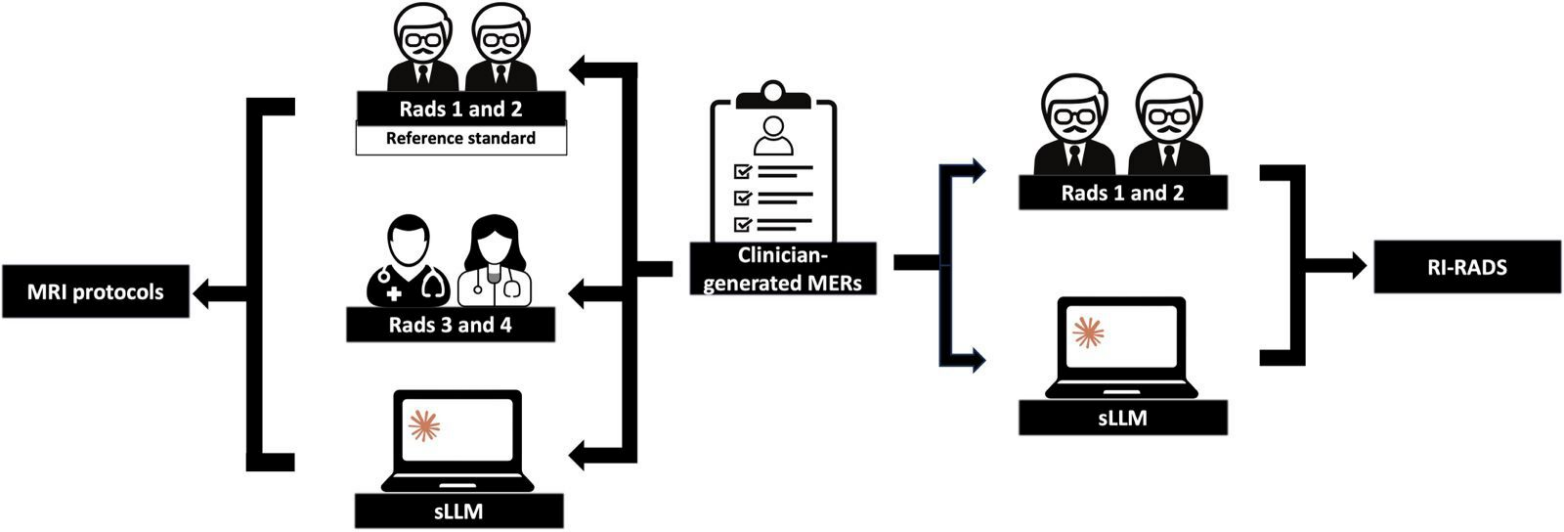
Study design flowchart. The initial clinician-generated MERs were extracted and then augmented using an sLLM. The clinician and sLLM MERs were compared for the quality of the clinical information available using the RI-RADS grading scale by 2 experienced radiologists (Rads 1 and 2). The MRI protocol accuracy for the sLLM and 2 board-certified radiologists (Rads 3 and 4, both with 2 years of experience) was determined by comparison against a reference standard provided by Rads 1 and 2. Claude version 3.5 (Anthropic) was used. *Clinical data included the last clinical entry and imaging reports (eg, computed tomography (CT) of the neck for an MRI nasopharynx). The sLLM and all board-certified radiologists had access to the MRI protocol guidance provided on the hospital intranet. MERs: MRI examination requests; MRI: magnetic resonance imaging; RI-RADS: Reason-for-Exam Imaging Reporting and Data System; sLLM: secure large language model.

Overall, 608 MRI examination requests were collected from 528 patients (mean 51.2 y, SD 19.2; range 4‐93 y). In total, 52.8% (279/528) patients were women and 47.2% (249/528) were men (Table 1). An additional 27 MRI examinations (26 patients) were excluded from analysis due to incomplete EMRs (eg, external referrals) (Figure 1).

**Table 1.** Patient and magnetic resonance imaging examination characteristics.

Characteristics	Values
Age (y), mean (SD; range)	
All (N=528)	51.2 (SD 19.2; 4-93)
Women (n=279)	52.9 (SD 17.4; 4-93)
Men (n=249)	49.3 (SD 21; 5-92)
MRI^a^ study specialties (N=608), n (%)	
Body MRI (n=206)	
Rectum/perineum fistula	28 (13.6)
Uterus/cervix cancer	26 (12.6)
Liver routine	21 (10.2)
Prostate routine	21 (10.2)
Enterography	16 (7.8)
Pancreas routine	14 (6.8)
Other	80 (38.8)
Musculoskeletal MRI (n=203)	
Lumbar spine	25 (12.3)
Cervical spine	24 (11.8)
Shoulder	29 (14.3)
Knee	27 (13.3)
Pelvis	11 (5.4)
Other	87 (42.9)
Neuroradiology MRI (n=199)	
Brain + contrast	40 (20.1)
Orbits	18 (9)
Skull base/temporal bones	15 (7.5)
Brain routine (noncontrast)	15 (7.5)
Brain stroke (acute)	14 (7)
Brain MR angiography	12 (6)
Pituitary dynamic	12 (6)
Oral cavity/neck	12 (6)
Nasopharynx and neck	10 (5)
Other	51 (25.6)
MRI study type (N=608), n (%)	
Routine (noncontrast)	239 (39.3)
Contrast	369 (60.7)
Contrast use by specialty	
Body MRI	Contrast 175 (85); noncontrast 31 (15)
Musculoskeletal MRI	Contrast 55 (27); noncontrast 148 (73)
Neuroradiology MRI	Contrast 139 (70); noncontrast 60 (30)

a MRI: magnetic resonance imaging.

Out of the 608 MRI examinations analyzed, there was the following subspecialty breakdown: 206 (34%) body, 203 (33%) musculoskeletal, and 199 (33%) neuroradiology. Contrast was administered in 85% (175/206) of body scans, 27% (55/203) of musculoskeletal scans, and 70% (139/199) of neuroradiology scans, yielding 60.7% (369/608) of contrast studies overall. Within each specialty, the most common protocols were rectum or perineal fistula, uterus or cervix cancer, liver, and prostate studies for body MRI; lumbar and cervical spine along with knee and shoulder examinations for musculoskeletal MRI; and postcontrast brain, orbital, and skull-base studies for neuroradiology MRI (Table 1).

### Adequacy of the Radiology Request Forms

RI-RADS gradings for the MERs were performed independently by 2 experienced radiologists (Rad 1 and Rad 2) (Table 2). Interobserver agreement (AC1) was almost perfect for the sLLM-augmented MERs (AC1 0.97, 95% CI 0.94‐0.99) and moderate for the clinician MERs (AC1 0.43, 95% CI 0.34‐0.52).

**Table 2.** Reason for exam imaging reporting and data system grades for the clinician and secure large language model–augmented magnetic resonance imaging examination requests^a^.

RI-RADS^b^ grade	Radiologist 1		Radiologist 2	
	Clinician MERs^c^ (n=608), n (%, 95% CI)	sLLM^d^ MERs (n=608), n (%, 95% CI)	Clinician MERs (n=608), n (%, 95% CI)	sLLM MERs (n=608), n (%, 95% CI)
A/B	484 (79.6, 95% CI 76.2‐82.8)	604 (99.3, 95% CI 98.3‐99.8)	583 (95.9, 95% CI 93.9‐97.3)	608 (100, 95% CI 99.51‐100)
C/D	124 (20.4, 95% CI 17.2‐23.6)	4 (0.7, exact 95% CI, Clopper-Pearson 0.2‐1.7)	25 (4.1, 95% CI 2.7‐6)	0 (0, exact 95% CI, Clopper-Pearson 0‐0.5)

a Values are the number of studies, with 95% CIs in brackets.

b RI-RADS: Reason-for-Exam Imaging Reporting and Data System.

c MERs: magnetic resonance imaging examination requests.

d sLLM: secure large language model.

Clinical information on the sLLM-augmented MERs was rated significantly higher than the original requests by both radiologists. For Rad 1, clinician MERs had 484/608 rated A/B (79.6%; 95% CI 76.2%-82.8%) and 124/608 rated C/D (20.4%; 95% CI 17.2%-23.6%), whereas sLLM MERs had 604/608 rated A/B (99.3%; 95% CI 98.3%-99.8%) and 4/608 rated C/D (0.7%; exact 95% CI, Clopper-Pearson, 0.2%-1.7%). For Rad 2, clinician MERs had 583/608 rated A/B (95.9%; 95% CI 93.9%-97.3%) and 25/608 rated C/D (4.1%; 95% CI 2.7%-6.0%), while sLLM MERs had all 608/608 rated A/B (100%; 95% CI 99.51%-100%) and 0/608 rated C/D (0%; exact 95% CI, Clopper-Pearson, 0%-0.5%).

No clinically significant hallucinations were identified in the sLLM outputs. Notably, the sLLM-generated MERs converted 28 spine MRI referrals (28/608, 4.6%) that were rated limited or deficient (7 of which omitted prior spinal surgery) into requests containing adequate clinical details.

### MRI Protocoling Accuracy Comparison

For protocoling, experienced radiologists 1 and 2 showed almost perfect agreement on the region (AC1 0.97, 95% CI 0.96‐0.99) and need for contrast (AC1 0.99, 95% CI 0.99‐1). A consensus protocol was determined for all 608 cases.

For all 608 MRI examinations, the sLLM matched the subspecialty reference standard in 566 (93.1%, 95% CI 89.6‐96.6) cases overall, compared with 556 (91.4%, 95% CI 87.6‐95.3) for Rad 3 and 560 (92.1%, 95% CI 88.4‐95.8) for Rad 4 (Table 3). This difference was not significant for the sLLM vs Rad 3 (*P*=.23) and Rad 4 (*P*=.40). Accuracy for region or coverage selection was similar across readers (sLLM: 579/608, 95.2%; Rad 3: 585/608, 96.2%; Rad 4: 573/608, 94.2%) with no significant differences between the sLLM vs Rad 3 (*P*=.46) and Rad 4 (*P*=.36). The sLLM, coupled with the use of the deterministic rules-based parsing script, demonstrated a slight advantage for contrast determination, being correct in 94.4% (574/608; 95% CI 91.3‐97.5) studies vs 92.1% (560/608; 95% CI 88.4‐95.8) for Rad 3 (*P*=.027) and 92.9% (565/608; 95% CI 89.4‐96.4) for Rad 4 (*P*=.16). Manual review of disagreements indicated that the sLLM correctly detected infection or inflammation in 2 cases (eg, suspected bursitis around the hip and shoulder), possible nerve lesions in 2 cases, and prior spine surgical details in 1 case, which were documented in the electronic record but not stated on the MER. These details triggered appropriate contrast recommendations that were omitted by at least 1 radiologist.

**Table 3.** Magnetic resonance imaging protocoling accuracy for the secure large language model and board-certified radiologists vs the reference standard.

MRI^a^ protocol vs reference standard	Radiologist 3	Radiologist 4	sLLM^b^
All MRI studies (N=608), n (%, 95% CI)			
Overall protocol correct	556 (91.4, 95% CI 87.6‐95.3)	560 (92.1, 95% CI 88.4‐95.8)	566 (93.1, 95% CI 89.6‐96.6)
Region correct	585 (96.2, 95% CI 93.6‐98.8)	573 (94.2, 95% CI 91.1‐97.4)	579 (95.2, 95% CI 92.3‐98.1)
Contrast decision correct	560 (92.1, 95% CI 88.4‐95.8)	565 (92.9, 95% CI 89.4‐96.4)	574 (94.4, 95% CI 91.3‐97.5)
Subspecialty subsets			
Musculoskeletal (n=203), n (%, 95% CI)			
Overall protocol correct	186 (91.6, 95% CI 87.8‐95.4)	180 (88.7, 95% CI 84.3‐93)	187 (92.1, 95% CI 88.4‐95.8)
Region correct	199 (98, 95% CI 96.1‐99.9)	182 (89.7, 95% CI 85.5‐93.8)	194 (95.6, 95% CI 92.7‐98.4)
Contrast decision correct	190 (93.6, 95% CI 90.2‐97)	185 (91.1, 95% CI 87.2‐95)	196 (96.6, 95% CI 94‐99.1)
Neuroradiology (n=199), n (%, 95% CI)			
Overall protocol correct	184 (92.5, 95% CI 88.8‐96.1)	189 (95, 95% CI 91.9‐98)	185 (93, 95% CI 89.4‐96.5)
Region correct	189 (95, 95% CI 91.9‐98)	194 (97.5, 95% CI 95.3‐99.7)	190 (95.5, 95% CI 92.6‐98.4)
Contrast decision correct	184 (92.5, 95% CI 88.8‐96.1)	189 (95, 95% CI 91.9‐98)	184 (92.5, 95% CI 88.8‐96.1)
Body (n=206), n (%; 95% CI)			
Overall protocol correct	186 (90.3, 95% CI 86.2‐94.3)	191 (92.7, 95% CI 89.2‐96.3)	194 (94.2, 95% CI 91‐97.4)
Region correct	197 (95.6, 95% CI 92.8‐98.4)	197 (95.6, 95% CI 92.8‐98.4)	195 (94.7, 95% CI 91.6‐97.7)
Contrast decision correct	186 (90.3, 95% CI 86.2‐94.3)	191 (92.7, 95% CI 89.2‐96.3)	194 (94.2, 95% CI 91‐97.4)

a MRI: magnetic resonance imaging.

b sLLM: secure large language model.

Within the musculoskeletal subset (N=203 examinations), overall agreement with the reference standard was 187 (92.1%, 95% CI 88.4‐95.8) for the sLLM, 186 (91.6%, 95% CI 87.8‐95.4) for Rad 3, and 180 (88.7%, 95% CI 84.3‐93) for Rad 4. These differences were not significant (*P*>.99 and *P*=.15 for sLLM vs Rad 3 and Rad 4, respectively). The sLLM matched Rad 3 for region accuracy (n=194, 95.6% vs 199, 98%; *P*=.23) and exceeded Rad 4 (n=182, 89.7%; *P*=.018) (Table 3). For contrast, coupled with the use of the deterministic rules-based parsing script, the sLLM was correct in 196 (96.6%, 95% CI 94‐99.1) studies, outperforming Rad 4 alone (n=185, 91.1%; *P*=.006) with no significant difference compared to Rad 3 (n=190, 93.6%; *P*=.099).

For body MRI (N=206 examinations), the sLLM had the highest overall accuracy (n=194, 94.2%, 95% CI 91‐97.4), although this was not significantly different vs Rad 3 (n=186, 90.3%; *P*=.089) and Rad 4 (n=191, 92.7%*; P*=.33). Review of body MRI discrepancies indicated that the sLLM recommended more focused uterus or cervix and MRI rectum and perineum protocols in 2 cases compared to a more general pelvis by the 2 radiologists. For contrast, the sLLM had the highest accuracy (n=194, 94.2%, 95% CI 91‐97.4), although this was not significantly different vs Rad 3 (n=186, 90.3%; *P*=.09) and Rad 4 (n=191, 92.7%; *P*=.33).

In neuroradiology MRI (N=199 examinations), Rad 4 achieved the highest overall accuracy (n=189, 95%, 95% CI 91.9‐98), although this was not significant vs the sLLM (n=185, 93%; *P*=.50) and Rad 3 (n=184, 92.5%; *P*=.71). Review of neuroradiology discrepancies indicated that the sLLM occasionally recommended skull-base protocols in 5 cases with suspected cranial nerve pathologies based on the clinical notes, whereas radiologists 3 and 4 accepted the original brain request when they deemed that coverage was adequate. For contrast, Rad 4 achieved the highest accuracy (n=189, 95%, 95% CI 91.9‐98), although this was not significant vs the sLLM (n=184, 92.5%; *P*=.30) and Rad 3 (n=184, 92.5%; *P*=.32) which were closely aligned.

## Discussion

### Principal Findings

In this study, we compared MERs provided by the referring clinician with those augmented by our institutional sLLM. Our results show that an sLLM can upgrade the clinical usefulness of MERs and aid protocol selection across body, neuroradiology, and musculoskeletal practice. After augmentation, fewer than 1% of requests (0%‐0.7%) were graded deficient or limited (RI-RADS C or D), compared with up to 1 in 5 (4.1%‐20.4%) of the clinician originals, and interreader agreement rose from moderate to almost perfect.

The sLLM protocoling accuracy was compared with that of 2 board-certified junior general radiologists. Overall protocol accuracy of the sLLM (566/608, 93.1%) was close to that of the 2 board-certified radiologists (Rad 3: 556/608, 91.4%; *P*=.23 and Rad 4: 560/608, 92.1%; *P*=.40), although the study was not powered to test statistical noninferiority or equivalence. Observed accuracy differences should not be interpreted as evidence of statistical similarity but instead as descriptive comparisons indicating that the sLLM operated within the performance range of general radiologists on this dataset.

For contrast decisions, the sLLM demonstrated accuracy of 94.4% (574/608), which was superior to Rad 3 (560/608, 92.1%; *P*=.027) and was not significantly different from Rad 4 (565/608, 92.9%; *P*=.16). Manual review showed that slightly increased accuracy for the sLLM was driven by EMR information (correct detection of prior surgery, infection, or tumor history) that did not appear on the MER yet was important for protocol selection. In our pipeline, the sLLM’s role was to extract and summarize these clinically relevant details, while the final region and contrast decisions were made by a deterministic rule-based script using predefined institutional criteria. Importantly, protocol selection and contrast determination are treated as separate decision steps. Protocol names reflect the anatomical region and clinical indication, while contrast administration may be modified when an explicit clinician request for noncontrast imaging or a documented contraindication is detected. In such cases, the default contrast setting associated with a protocol may be overridden to preserve clinician intent and patient safety. As such, the observed accuracy reflects improved information extraction feeding into consistent rules, rather than autonomous clinical reasoning by the sLLM.

In one of this study’s cases, the clinician requested noncontrast MRI for cervical cancer recurrence due to renal impairment of estimated glomerular filtration rate 31‐48, although modern guidelines may permit the use of group II gadolinium agents at this level. We acknowledge that prioritizing clinician intent in such cases may risk suboptimal diagnostic utility and potential recall.

Most mismatches against the reference standard occurred in borderline cases where more than one subspecialty-specific protocol was reasonable. In body MRI, this typically involved the sLLM proposing a general pelvis study when radiologists selected a focused protocol such as prostate, uterus or cervix, or rectum. In spine MRI, a small number of discrepancies arose from consolidating concurrent cervical and lumbar requests into a single-region study. In neuroradiology, differences usually reflected choosing between brain vs skull base or temporal bone coverage in suspected cranial nerve pathology. These patterns suggest that minor refinements to protocol-selection rules could help reduce discrepancies.

### Comparison to Prior Work

These findings are consistent with recent evaluations of LLMs for noninterpretive tasks in radiology and add multisubspecialty evidence. Prior institutional studies using an sLLM for spine and musculoskeletal MRI reported similar gains, with musculoskeletal protocoling accuracy reaching 96% and exceeding 2 general readers at 88% and 89% [17,18]. In another recent study, Çamur et al [25] showed that 4 LLMs have strong potential for selecting appropriate imaging modalities. The 4 LLMs were tested on 240 clinical cases (120 ACR Appropriateness Criteria and 120 realistic scenarios) and compared their choices with 4 clinicians and 4 radiologists. The best model picked the correct imaging test in 98.3% (236/240) of ACR cases, matched a junior radiologist on realistic cases, gave identical answers across prompts (κ=1), and showed moderate to good reproducibility over time (κ~0.77‐0.89 short term; 0.51‐0.79 long term) [25]. Similarly, for CT protocol assignment, a fine-tuned support tool picked the right protocol on the first choice 92.3% of the time and within its top two 96.3% of the time, with an average processing time of less than 1 second per case. When clinicians used it, resident accuracy improved from 0.913 to 0.936 with a 14% reduction in reading time, and attending accuracy increased from 0.920 to 0.926 with a 12% time saving [26]. In musculoskeletal MRI, a GPT-4 system linked to a small knowledge base reached 92.86% accuracy on ACR-based cases, outperformed a baseline model and standard GPT-4, matched most subspecialists, and was better at flagging when the clinical information was insufficient, which mirrors our MER enrichment step [27]. Beyond protocol choice, a radiology operations study showed GPT-4 routed 96% of in-scope procedure requests and 76% of out-of-scope requests correctly at a cost of approximately US $0.03 per request, indicating potential for increased efficiency and cost benefits for routing workflows [28]. Together with our results, these data suggest that institution-hosted sLLMs could aid improvements in clinical context, standardize protocol decisions, reduce unnecessary contrast and radiation, and potentially save time and costs by reducing protocoling time and reschedules.

### Limitations and Future Directions

Our study has several limitations. First, this was a single-center, retrospective evaluation involving only outpatient MERs. The sLLM pipeline relied on the specific institutional MRI protocol repository and access to the EMR structure, and the realized gains may differ in institutions using different order-entry systems or protocol libraries. Future work should include prospective, multicenter evaluations that measure operational outcomes (time to scan, rescheduling, repeat imaging), cost-effectiveness analyses that incorporate staff time and scanner utilization, and extension of the sLLM pipeline to CT and ultrasound.

Second, some patients contributed multiple examinations, and the dataset lacked patient-linked identifiers; therefore, cluster-robust or mixed-effects analyses were not feasible. Results should be interpreted at the examination level, with future work planned to incorporate patient-level clustering.

Third, formatting differences between the clinician and augmented MRI examination requests could have partially unblinded the graders, with the potential to artificially inflate the sLLM’s RI-RADS scores, although the direction of any resulting bias is uncertain. In our study, the output format (subheadings; eg, clinical history, reason for exam) was identical for the clinician and sLLM forms, although differences in language and style may have unblinded the reviewers. This is difficult to rectify but could be addressed in future studies using standardized outputs.

Fourth, the exclusion of cases with incomplete electronic records introduces a potential selection bias, as these omitted cases may represent some of the more challenging instances for protocoling. In addition, the manual extraction of the latest relevant clinical entry and pertinent prior imaging reports without an automated failsafe mechanism provided a methodological simplification, which is different from real-life human EMR review, which involves searching through all relevant notes and prior imaging. Together, these could potentially inflate the sLLM’s protocoling accuracy and are an important area for future improvement. Future scaling may also benefit from approaches such as hierarchical summarization or RAG-style chunking for very large EMR entries.

Fifth, we assessed board-certified radiologists who demonstrated high accuracies for protocoling (>91.4%), leaving little room for the sLLM to improve on this. LLM assistance for less experienced readers, including technologists, could provide more value and will need to be assessed. Nonetheless, even if accuracy gains over experts are marginal, the standardization benefit and fatigue reduction offered by the sLLM support its deployment. A further improvement may include an “assisted radiologist with sLLM” arm to separate automation from augmentation.

Sixth, although the study included more than 600 MRI examinations, the sample size was powered for detecting a 15% difference in clinical-information adequacy, not for establishing noninferiority or equivalence in protocol accuracy. The small absolute differences observed between the sLLM and board-certified radiologists (eg, 93.1% vs 91.4%; *P*=.23) therefore cannot be interpreted as statistical equivalence.

Seventh, we deliberately confined the evaluation to MRI, which is our most protocol-intensive modality and the largest component of departmental workload, but acknowledge that CT and ultrasound will require separate validation prior to broader application.

Eighth, the review for clinically significant hallucinations was limited to discordant cases, and there is a theoretical risk that the sLLM could generate benign or “silent” hallucinations in the clinical summary that do not alter the protocol decision but could still degrade the medical record.

Finally, although no clinically significant hallucinations were observed, it is well known that LLMs are prone to producing factual errors that could have major clinical implications [29-31]. In addition, recent work shows that LLMs can be vulnerable to adversarial hallucination in clinical decision-support settings, with mitigation prompts only partially reducing errors and lower temperature settings providing no meaningful benefit [31]. Continuous monitoring and postdeployment guardrails therefore remain essential, but were beyond the scope of this study.

### Conclusion

In this multisubspecialty MRI cohort, an sLLM improved MRI examination request completeness and demonstrated protocol accuracy comparable to experienced radiologists; however, the study was not powered to establish noninferiority or equivalence for protocol accuracy.

The end-to-end sLLM pipeline, consisting of request form enrichment with EMR data and a rule-based protocoling technique, offers a practical pathway for more efficient, standardized protocoling while reducing the administrative burden on clinicians.

## Supplementary material

10.2196/82579 Multimedia Appendix 1 Large language model prompts.

10.2196/82579 Multimedia Appendix 2 Reason for Exam Imaging Reporting and Data System (RI-RADS).

## Abbreviations

EMR: electronic medical record
LLM: large language model
MERs: MRI examination requests
MRI: magnetic resonance imaging
RAG: retrieval-augmented generation
RI-RADS: Reason-for-Exam Imaging Reporting and Data System
sLLM: secure large language model
