# Supplementary material for: Initial Insights Into an Institutional Secure Large Language Model for Magnetic Resonance Imaging Examination Requests: Retrospective Study
Source: J Med Internet Res. 2026 Apr 7;28:e82579. doi: 10.2196/82579 (PMC13055936; doi:10.2196/82579)
Supplement: Multimedia Appendix 1 [file jmir-v28-e82579-s001.docx]

1. Large Language Model Prompts

**Part 1: Augmentation of the original request form**

Note: elements in {} are dynamically generated from the request form and clinical notes.

<request_form>{request_form }</request_form>

<clinical_notes>{notes}</clinical_notes>

Instruction:

Note: The sLLM extracts and organises clinical information; final protocol rules are applied later by a separate deterministic script.

Task 1: Lift relevant information from request form and clinical notes

a) Reason for {mri_exam} and relevant medical history

Information from request form: According to the request form, what are the suspected diagnoses or reasons for the {mri_exam}? What was the contrast specified for the {mri_exam}?

Details and history from clinical notes: Lift all text from the clinical notes that support the information from request form, provide context for the {mri_exam} request or indicate the plan for {mri_exam}. This includes all medical or surgical history, past and present complaints (include all information about the chronicity and location of the symptoms), observations, pertinent laboratory or imaging findings and suspected diagnoses which would give context for the {mri_exam}.

b) Patient MRI risks

Lift text from the request form and clinical notes which indicate any of the following

i) MRI contrast agent contraindications such as contrast agent allergy, kidney disease, renal impairment or pregnancy

ii) Recent surgery or surgical history relevant to the MRI exam

iii) MRI contraindications or metal implants. History of ACDF, TLIF, laminoplasty or laminectomy indicates possible spinal instrumentation.

Task 2: {mri_exam} summary

Fill in the following markdown summary table for the {mri_exam} request. Do not include exams irrelevant to the request.

Each row must have the following fields:

- Exam ordered

- Contrast requested: Contrast/Non-contrast/Unspecified

- Region

- Side: Left/Right. Leave blank if not applicable

- Clinical Findings: Elaborate on symptoms, complaints or observations relevant to MRI exam region that justifies the {mri_exam}. Include any information on the location and chronicity of the symptoms. Leave blank if not applicable, do not make up facts.

- Suspected diagnosis: Differential or suspected diagnoses relevant to the {mri_exam}

- Reason: Objective of the {mri_exam} or questions the {mri_exam} can answer.

- Clinical Notes Discrepancy: Indicate yes if the {mri_exam} is not supported by details from the clinical notes. Also indicate yes if the request reason or suspected diagnosis in the form is inconsistent with the clinical notes. Answer in the format, Yes/No; details if yes

- Contrast Risk: Details of any risks or contraindications for MRI contrast agents, such as allergy to contrast agents, pregnancy or renal impairment. Only include factors explictly mentioned in the clinical notes. Separate multiple records with comma, do not generate extra columns. Indicate None if no allergy or renal impairment is mentioned.

- Past Surgery: Surgical history relevant to the {mri_exam}. In the format, Date; Surgery region; Surgery details. Separate multiple records with comma, do not generate extra columns. Leave blank if no prior surgery is mentioned.

- Instrumentation or Implant (safety guardrail): Details of any instrumentation or implant that is MRI incompatible or an MRI risk, eg, pacemaker, metal implants, etc. Leave blank if patient has no instrumentation or implant.

Note: Importantly, institutional safeguards already exist to flag pacemakers, implants, and MRI contraindications. At least once during exam request submission and again during the patient interview prior to scanning.

Note: The sLLM is not allowed to infer details. Only information explicitly present in the request form or clinical notes is returned.

**Part 2: Determination of Protocol and Contrast Requirements**

Note: The body and neuro MRI contrast requirements are largely determined by the selected protocol, unless the patient has contra-indications for contrast. MSK has finer contrast requirements depending on the reason for request.

In this step, the sLLM provides the structured summary, while the final exam protocol and contrast classification follow predefined institutional rules implemented by a parsing script

Importantly, protocol selection and contrast determination are treated as separate decision steps. Protocol names reflect the anatomical region and clinical indication, while contrast administration may be modified when an explicit clinician request for non-contrast imaging or a documented contraindication is detected. In such cases, the default contrast setting associated with a protocol may be overridden to preserve clinician intent and patient safety.

**Protocol Determination Prompt**

<request_form>{request_form}</request_form>

<summary>{summary from part1}</summary>

<protocol-details>{Table of MRI protocol institutional guidelines }<protocol-details>

Based on the summary of the MRI request and MRI protocol details, is the {mri_exam} ordered the most appropriate exam to be performed?

Give your answer in a table, where each row has the following fields:

- Exam ordered

- Clinical Findings: Symptoms, complaints or observtions relevant to the {mri_exam}

- Exam Reason: Objective of the {mri_exam}, suspected diagnoses or questions the {mri_exam} can answer

- Contrast Risk*: Details of any contrast risks or contraindications such as MRI contrast allergy, ongoing pregnancy at time of exam or renal impairment. Indicate None if not applicable.

- Exam Protocol: Most appropriate MRI exam given the MRI exam reason. If appropriate, multiple non-overlapping protocols may be listed separated by ;

- Exam Contrast: Contrast or Non-contrast. If there are multiple protocols, list in order of protocol, separated by ;

- Exam Coverage: MRI exam region of interest

- Protocol Discrepancy: Indicate yes if {mri_exam} is inappropriate for the exam reason. In the format, Yes/No; details if yes

*note: The actual eGFR threshold was not included in the original prompt. We observed that it was able to identify renal impairment as eGFR < 60, as Claude 3.5 had been trained on multiple texts including medical texts.

**Contrast Determination (MSK only)**

Contrast for MSK studies was based on a rules-based system based on the presence of contrast factors in the augmented request form and identified contrast contra-indications. The sLLM does not choose contrast but supplies the extracted elements used by the script.

Based on the summary of the MRI request and relevant clinical history, is any of the following conditions the among the suspected diagnoses or reason for the MRI exam request?

i) Lesion or Tumor

- Metastases or known history of malignancy, cancer or neoplastic disease

- Lumps, masses or fibromas

- Lipomas or lipomatous lesions

- Vascular malformations or lesions

- Bone and soft tissue lesions or tumors at all sites. These include nerve sheath tumors, neuromas or schwannomas, and bone tumors such as osteosarcoma, chondrosarcoma or chondroid lesions, suspected sarcoma, etc. This does not include osteochondral lesions, also known as osteochondritis dissecans.

- Spinal cord tumor or intramedullary lesion

- Pigmented villonodular synovitis (PVNS) or tenosynovial giant cell tumor (TSGCT)

ii) Infection or Inflammation

- Osteomyelitis, septic arthritis, spondylodiscitis, discitis, collection, abscess, bone infection, soft tissue infection, myelitis, meningitis, phlegmon, epidural disease, etc

- Immune-mediated inflammatory conditions such as inflammatory arthritis, rheumatoid arthritis, psoriatic arthritis, systemic lupus erythematosus (SLE), lupus Arthritis, juvenile idiopathic arthritis (JIA), still's disease, reactive arthritis, Reiter's syndrome, post-infectious arthritis, seronegative rheumatoid arthritis, Guillain-Barré Syndrome (GBS) or other inflammatory arthropathy

- Enteropathic arthritis such as Inflammatory Bowel Disease (IBD)-Associated arthritis, Crohn's-Associated arthritis or Ulcerative Colitis-Associated arthritis

- Spondyloarthropathies such as axial spondyloarthritis, spondylitis, ankylosing spondylitis (AS), spondyloarthritis (SpA), sacral iliitis, sacroiliitis, SI joint inflammation, SI arthritis or SI arthropathy

- Overlap syndrome, Mixed Connective Tissue Disease (MCTD)

- Scleroderma or polymyalgia rheumatica

- Myositis, polymyositis, dermatomyositis, inclusion body myositis (IBM), juvenile myositis, necrotizing autoimmune myopathy

- Vasculitis-Associated arthritis, giant cell arteritis (GCA) or granulomatosis with polyangiitis (GPA)

- Tenosynovitis, synovitis, bursitis, osteitis or enthesitis

- Gout, uric acid arthritis or gouty arthritis

- Crystal arthropathy such as pseudogout, chondrocalcinosis, calcium pyrophosphate dihydrate (CPPD) arthritis or calcific tendonitis.

- Differential diagnosis or suspicion of inflammation or inflammatory conditions

- Note: Do not include epicondylitis, spondylosis, osteoarthritis or any other degenerative arthritis. Do not include injury, tenderness due to injury, dislocation, fractures, tears or deformities. Do not include non-inflammatory swelling or pain like tendinopathy.

iii) Nerve Injury

- Neuropathy or mononeuritis multiplex

- Axonotmesis or neurotmesis

- Nerve entrapment, pronator teres syndrome, or plexopathy

- Note: Do not include numbness, nerve root injury such as radiculopathy or spinal nerve compression.

iv) Spine instrumentation

- Recorded or likely spinal instrumentation (history of ACDF, TLIF, laminoplasty, laminectomy, spinal fusion, decompression, etc) in the exam region

- Do not include planned procedures

v) Recent spine operation

- Post-operative assessment to differentiate between scar tissue and recurrent disc herniation. Also to assess for any collection

- Do not include planned surgery

- Spine operation in the past 5 years, between {year-5} and {year}

Only include reasons and conditions explicitly mentioned in the summary, do not speculate, or make your own inferences.

Give your answer in a table, where each row has the following fields:

- Exam ordered

- Exam Reason: Objective of the MRI exam, suspected diagnoses or questions the MRI exam can answer

- Lesion or Tumor: Based on the exam reason and clinical history relevant to the exam objective, in the format, Yes/No; details if yes.

- Infection or Inflammation: Based on the exam reason and clinical history relevant to the exam objective, in the format, Yes/No; details if yes. Do not include swelling, pain or tenderness.

- Nerve Injury: Based on the exam reason and clinical history relevant to the exam objective, in the format, Yes/No; details if yes.

- Spine Instrumentation: In the format, Yes/No; details if yes.

- Recent Spine Operation: In the format, Yes/No; details if yes.

1. MRI Protocol Institutional Guidelines

**Body**

| **Protocol** | **Contrast** | **Indications** | **Comments** |
| --- | --- | --- | --- |
| MRI Liver, Routine | Contrast | - All liver related except for indications for Primovist - Can be done for FNH follow up too | Standard contrast with extracellular agents such as Dotarem, Gadovist, Clariscan |
| MRI Liver, Primovist | Contrast (Primovist) | - FNH characterisation - suspected post-op bile leak / biloma - NASH - Specified by clinicians (e.g. HCC, mets) | Caution: hyperbilirubinemia / jaundice (high Bilirubin affects Primovist uptake) Caution against giving Primovist for diagnosis of haemangioma |
| MRI Liver, Donor workup | Contrast | Donor liver pre-transplant workup | Standard protocol uses Primovist contrast |
| MRI Liver, Iron Quantification | Non-contrast | Assessment of iron overload, thalassemia | Done in GE scanner with IdealIQ sequences (T2*, R2* maps) |
| MRI Elastography (Liver) | Non-contrast | Assessment of liver stiffness in fibrosis and cirrhosis, NASH | Selected scanners only Note: Fasting required |
| MR Cholangiopancreatography (MRCP) | Non-contrast | - Gallstones - Biliary tree dilatation - Pancreatic cystic lesions / IPMN follow up | If clinician request for MRCP with contrast in cases of suspected bile leak, to do MRI liver with Primovist |
| MRI Pancreas, Routine | Contrast | - Pancreatic cystic lesions / IPMN - Pancreatitis - Pancreatic malignancy | Standard protocol has no Ax LAVA 5 min delayed +C unlike MRI liver |
| MRI Adrenal Glands, Routine | Contrast | Characterisation of adrenal nodule |  |
| MRI Kidneys, Routine | Contrast | - Renal cysts - Renal neoplasms |  |
| MRI Kidneys, Volumetric | Non-contrast | Polycystic kidneys volume |  |
| MRI Urogram, Routine | Contrast | - Evaluation of complex renal and urinary tract pathology - Urinary tract obstruction | Optional: post-micturition phase (if suspected urethral diverticulum) Caution: For evaluation of pathology limited to the urinary bladder and urethra, consider doing MRI Pelvis |
| MRA Renal Arteries, Routine | Contrast | Renal artery stenosis | Optional: Non-contrast |
| MRI Prostate, Routine | Contrast | - Raised PSA - Cancer staging - Suspected local recurrence post- prostatectomy | Typically done in 3T (unless cardiac pacemaker, done 1.5T instead)  Bi-parametric may be considered in low eGFR |
| MRI Rectum and Perineum, Cancer | Non-contrast | Rectal cancer staging | Might be done with contrast for delineating anal in anal cancer |
| MRI Rectum and Perineum, Fistula | Contrast | Perianal fistula / collection |  |
| MRI Enterography, Routine | Contrast | - Inflammatory bowel disease - Suspected Small bowel lesions | Non-contrast MRE has no clinical utility and may be cancelled |
| MRI Uterus / Cervix, Cancer | Contrast | - Uterine mass - Uterine Cancer staging | Standard protocol DCE done sagittal |
| MRI Uterus / Cervix, Endometriosis | Contrast | Endometriosis |  |
| MRI Uterus / Cervix, Ovarian | Contrast | - Ovarian / adnexal mass - Ovarian Cancer staging |  |
| MRI Uterus / Cervix, Placenta | Non-contrast | Placenta accreta/increta/percreta |  |
| MRI Uterus / Cervix, Mullerian anomaly | Non-contrast | Mullerian duct anomaly |  |
| MRI Abdomen, Routine | Contrast | - Cancer screening - Evaluation of abdominal mass | Optional: DWI/ADC |
| MRI Abdomen-Pelvis, Routine | Contrast | - Cancer screening - Evaluation of abdominal and pelvic masses | Optional: DWI/ADC |
| MRI Pelvis, General | Contrast | - Cancer screening - Evaluation of non-MSK related pelvic mass | Optional: DWI/ADC |
| MRA Whole Aortogram, Routine | Contrast | - Aortic aneurysm - Aortic dissection follow-up - Marfan syndrome |  |
| MRA Whole Aortogram, Takayasu arteritis | Contrast | Takayasu arteritis | Standard protocol has DB sequence for selected level |
| MRA Thoracic Aortogram, Routine | Contrast | - Aortic aneurysm - Aortic dissection follow-up | Avoids radiation from CTA |
| MRA Abdominal Aortogram, Routine | Contrast | - Aortic aneurysm - Aortic dissection follow-up | Avoids radiation from CTA |
| MRI Mediastinum, Routine | Contrast | Mediastinal mass characterisation | Optional: DWI/ADC |
| MRI Breast Bilateral, Routine | Contrast | Breast cancer screening |  |
| MRI Breast Bilateral, Non-Contrast | Non-contrast | Only if contraindicated for contrast, or specifically requested |  |
| MRI Fetal Scan | Non-contrast | Always non-contrast |  |

**Neuro**

| **Protocol** | **Contrast** | **Indications** | **Comments** |
| --- | --- | --- | --- |
| MRI Brain, Routine | Non-contrast | Headache, migrane [sic] - Mental status changes, diplopia - Trauma | MRI Brain, Dementia should be done instead of routine MRI Brain if dementia is suspected |
| MRI Brain, With Contrast | Contrast | - Brain lesions, cancer, tumors or metastases, including epileptogenic lesions - Infection or Inflammation - Demyelinating diseases like multiple sclerosis (MS) - Aneurysm, AVM or vasculitis - Post-surgery or Post-radiotherapy follow-up |  |
| MRI Brain, Dementia | Non-contrast | Dementia | MRI brain with an additional sequence for dementia |
| MRI Brain, Epilepsy | Non-contrast | Seizure, epilepsy |  |
| MRI Brain, Stroke Screen | Non-contrast | Stroke Screen | - MRI brain stroke screen sequences with MRA Brain - Strictly non-contrast. If contrast is needed, or patient has a history of vasculitis, an MRI brain with contrast and MRA Brain should be done instead |
| MRA Brain | Non-contrast | Aneurysm, AVM, post-coiling | Angiogram of the circle of Willis. Should be done concurrently with other relevant brain studies |
| MRV Brain | Non-contrast | Sinus thrombosis, Venous infarct | Please note that this should be performed with a concurrent or recent MRI brain with contrast |
| MRI Brain, Stereotaxy | Contrast | Preoperative planning |  |
| MRI Brain, Perfusion | Contrast | Stroke, Tumor |  |
| MRI Brain, Spectroscopy | Non-contrast | Tumor vs post RT necrosis | Patient must be able to keep still Not suitable for lesions near bony structure, air and CSF and at least 1 cm in size |
| MRI Orbits / Anterior Visual Pathway, Routine | Contrast | - Orbital or ocular mass lesions - Optic neuritis - Proptosis/Thyroid eye disease Visual disturbance - Retinoblastoma | Any concurrent brain study should also be done with contrast |
| MRI Orbits / Anterior Visual Pathway, Non-Contrast | Non-contrast | Only if contraindicated for contrast, or specifically requested |  |
| MRI Internal Acoustic Meatus, Screen | Non-contrast | For sensorineural hearing loss or suspected vestibulocochlear schwannoma | If a cause in the brain is considered, a concurrent MRI brain may be ordered |
| MRI Internal Acoustic Meatus, With Contrast | Contrast | - If there is a known tumour (e.g., known schwannoma or follow-up) - Infection or Inflammation. Example: labyrinthitis - Post-surgical follow-up or radiotherapy follow-up (e.g., acoustic neuromas) - Assessment of cochlear implant | MRI Skull Base & Temporal Bone is a better option for facial nerve or trigeminal nerve pathology. |
| MRI Internal Acoustic Meatus, With Delayed Contrast | Contrast | Cholesteatoma |  |
| MRI Skull Base & Temporal Bone, Routine | Contrast | - Any cranial nerve pathology. Olfactory nerve should be non-contrast unless specifically requested - TMJ infection - Recurrent cholesteatoma | SNHL or vestibulocochlear nerve alone should be considered for MRI Internal Acoustistic [sic] Meatus instead If a cause in the brain is considered, a concurrent MRI brain may be ordered |
| MRI Skull Base & Temporal Bone, Non-contrast | Non-contrast | - CN I (Olfactory nerve) - TMJ or bite issues not due to infection, inflammation or tumours |  |
| MRI Pituitary, Routine (Dynamic Contrast) | Contrast | - Pituitary neuroendocrine tumours, macroadenomas and microadenomas - Sellar or supra-sellar masses - Precocious puberty, SIADH | May be ordered with a concurrent MRI Brain |
| MRI Pituitary, Non-contrast | Non-contrast | Only if contraindicated for contrast, or specifically requested |  |
| MRI Salivary Glands, Routine | Contrast | Salivary gland tumours, lesions or inflammation |  |
| MRI Salivary Glands, Non-contrast | Non-contrast | Only if contraindicated for contrast, or specifically requested |  |
| MRI Intracranial Vessel Wall, Routine | Contrast |  |  |
| MRI Intracranial Vessel Wall, Non-Contrast | Non-contrast | Only if contraindicated for contrast, or specifically requested |  |
| MRI Nasal Cavity & Paranasal Sinuses, Routine | Contrast | Sinonasal pathology (tumors, infections, CSF leaks) |  |
| MRI Nasal Cavity & Paranasal Sinuses, Non-Contrast | Non-contrast | Only if contraindicated for contrast, or specifically requested |  |
| MRI Nasopharynx & Neck, Routine | Contrast | Nasopharyngeal carcinoma, lymph nodes and deep neck spaces, Larynx or thyroid lesion |  |
| MRI Nasopharynx & Neck, Non-Contrast | Non-contrast | Only if contraindicated for contrast, or specifically requested |  |
| MRI Oral Cavity Oropharynx & Neck, Routine | Contrast | Oral cancers, salivary gland lesions, and tongue pathologies |  |
| MRI Oral Cavity Oropharynx & Neck, Non-Contrast | Non-contrast | Only if contraindicated for contrast, or specifically requested |  |

**MSK**

| **Protocol** | **Contrast** | **Indications** | **Comments** |
| --- | --- | --- | --- |
| MRI, Cervical Spine, Routine | Non-contrast | Myelopathy, Radiculopathy, Cord compression or trauma, Pain, numbness, tingling, weakness in arms/shoulder/neck, Chronic nervous system disease | Covers ponto-medullary junction to T2 |
| MRI, Cervical Spine, With Contrast | Contrast | Tumor or cord lesion, Metastases (with cancer history), Infection (osteomyelitis, spondylodiscitis, discitis, etc.), Spinal Meningitis/TB, Multiple Sclerosis plaques (with demyelination history) | Post-operative cases within 5 years require contrast. |
| MRI, Thoracic Spine, Routine | Non-contrast | Myelopathy, Radiculopathy, Cord compression or trauma, Pain, numbness, tingling, weakness in arms/shoulder/neck, Chronic nervous system disease | Covers C7-L1 |
| MRI, Thoracic Spine, With Contrast | Contrast | Tumor or cord lesion, Metastases (with cancer history), Infection (osteomyelitis, spondylodiscitis, discitis, etc.), Spinal Meningitis/TB, Multiple Sclerosis plaques (with demyelination history) | Post-operative cases within 5 years require contrast. |
| MRI, Lumbar Spine, Routine | Non-contrast | Myelopathy, Radiculopathy, Cord compression or trauma, Pain, numbness, tingling, weakness in arms/shoulder/neck, Chronic nervous system disease | Covers T12-Sacrum |
| MRI, Lumbar Spine, With Contrast | Contrast | Tumor or cord lesion, Metastases (with cancer history), Infection (osteomyelitis, spondylodiscitis, discitis, etc.), Spinal Meningitis/TB, Multiple Sclerosis plaques (with demyelination history) | Post-operative cases within 5 years require contrast. |
| MRI, Lumbar Spine & SI Joints | Non-contrast (unless specified) | - Ankylosing spondilitis [sic] - Radiculopathy + Sacroiliitis | Covers T11-Coccyx Always non-contrast unless specifically requested by clinician. Is a different study from MRI, Lumbar Spine. |
| MRI, Sacrum & Coccyx, Routine | Non-contrast | Pain, Fracture, Sacroiliitis/sacro-iliac joint arthritis | Covers L5-Coccyx |
| MRI, Sacrum & Coccyx, With Contrast | Contrast | Osteomyelitis/infection/septic arthritis, Sacral sore, Tumor, Metastases (cancer history), Infection |  |
| MRI, Whole Spine, Routine | Non-contrast | - Myelopathy, Radiculopathy, Cord compression or trauma, Pain, numbness, tingling, weakness in arms/shoulder/neck, Chronic nervous system disease - Bone marrow screening -Congenital abnormalities of Spinal curvature (Scoliosis and kyphosis) | This is a screening MRI order, do not suggest as an alternative to other spine exams. Axial cuts will not necessarily cut parallel to the endplates for every level Coverage: Cervical Spine-Coccyx |
| MRI, Whole Spine, With Contrast | Contrast | Tumor or Cord lesion, Metastases (with cancer history), Infection (osteomyelitis, spondylodiscitis, discitis, etc.), Spinal Meningitis/TB, Multiple Sclerosis plaques (with demyelination history), Leptomeningeal disease, Post-op, Neurofibroma | This is a screening MRI order, do not suggest as an alternative to other spine exams. Post-operative cases within 5 years require contrast. |
| MRI Brachial Plexus, Routine | Non-contrast | Brachial plexopathies, Trauma or Impingement | Covers C3-Axilla |
| MRI Brachial Plexus, With Contrast | Contrast | Brachial plexus lesions (esp. secondary to cancer of breast and bronchus), tumors, infection, inflammation. |  |
| MRI Lumbar Plexus, Routine | Non-contrast | - Plexopathy - Piriformis Syndrome | Covers T12-Lesser trochanter Consider ordering MRI Lumbar Spine first if no prior MRI imaging is available |
| MRI Lumbar Plexus, With Contrast | Contrast | Lumbar/Lumbosacral plexus lesions |  |
| MRI Chest Wall, Routine | Non-contrast | - Muscle/tendon abnormalities of chest - Clavicle imaging - Ribs imaging - Sternum imaging - Sternoclavicular joints (SCJ) imaging |  |
| MRI Chest Wall, With Contrast | Contrast | - Bony and soft tissue lesions - Inflammation / Infection such as septic arthritis |  |
| MRI, Shoulder, Routine | Non-contrast | Shoulder pain/impingement, Rotator cuff tear, Labral tear, Frozen shoulder | Covers shoulder joint and surrounding tissues |
| MRI, Shoulder, With Contrast | Contrast | Bony/soft tissue lesions, Inflammation (synovitis, myositis), Infection (osteomyelitis, cellulitis, septic arthritis), Pigmented villonodular synovitis (PVNS) or tenosynovial giant cell tumor (TSGCT) | For inflammatory, infectious, or tumorous conditions |
| MRI, Shoulder, Arthrogram | Intra-articular contrast (fluoroscopy-guided) | On clinician request only: Recurrent dislocation, Labral tear, SLAP lesion | No intravenous contrast is required. Contrast will be injected directly into the joint under fluoroscopic guidance. |
| MRI, Arm, Routine | Non-contrast | Deformity, Muscle tears, Compression neuropathies not due to tumours | Covers whole humerus |
| MRI, Arm, With Contrast | Contrast | Bony/soft tissue lesions, Inflammation, Infection, PVNS/TSGCT |  |
| MRI, Forearm, Routine | Non-contrast | Deformity, Muscle tears, Compression neuropathies not due to tumours | Covers whole forearm |
| MRI, Forearm, With Contrast | Contrast | Bony/soft tissue lesions, Inflammation, Infection, PVNS/TSGCT |  |
| MRI, Elbow, Routine | Non-contrast | Muscle tears, Osteochondral defects, Loose bodies, Ulnar nerve compression | Covers elbow joint and surrounding tissues |
| MRI, Elbow, With Contrast | Contrast | Bony/soft tissue lesions, Inflammation, Infection, PVNS/TSGCT |  |
| MRI, Wrist, Routine | Non-contrast | Ligament/tendon assessment (TFCC, LT, SL), AVN scaphoid, Carpal Tunnel Syndrome, De Quervain’s tenosynovitis, Pain | Covers wrist joint and surrounding structures |
| MRI, Wrist, With Contrast | Contrast | Bony/soft tissue lesions, Inflammation, Infection, PVNS/TSGCT |  |
| MRI, Hand, Routine | Non-contrast | Ligament/tendon assessment, Arthritis, Pain | Can be subdivided into hand, finger, or thumb. |
| MRI, Hand, With Contrast | Contrast | Bony/soft tissue lesions, Inflammation, Infection, PVNS/TSGCT |  |
| MRI, Finger/Thumb, Routine | Non-contrast | Ligament/tendon assessment, Arthritis, Pain | Additional digits incur extra charges. |
| MRI, Finger/Thumb, With Contrast | Contrast | Bony/soft tissue lesions, Inflammation, Infection, PVNS/TSGCT | Additional digits incur extra charges. |
| MRI Pelvis, MSK | Non-contrast | Fracture(s) of the pelvic bone, Inguinal Hernia | Covers Iliac crest to lesser trochanter |
| MRI Pelvis, MSK With Contrast | Contrast | Musculoskeletal Infection, Metastases, Tumor | If sacroiliitis suspected, use MRI Sacrum & Coccyx instead |
| MRI Pelvis, General | Contrast | - Cancer screening - Pelvic mass for Evaluation | Optional: DWI/ADC |
| MRI, Hip, Routine | Non-contrast | AVN, Femoral Acetabular Impingement (FAI), Fracture, Labral tear (<60yo), Muscle tear, Sports hernia | Covers hip joint and its surrounding structures |
| MRI, Hip, With Contrast | Contrast | Bony/soft tissue lesions, PVNS, Inflammation (synovitis, myositis), Infection (osteomyelitis, cellulitis, septic arthritis) |  |
| MRI, Hip, Arthrogram | Intra-articular contrast (fluoroscopy-guided) | On clinician request only: FAI, Labral tear | Two studies required: fluoroscopy followed by MRI |
| MRI, Thigh, Routine | Non-contrast | Pain, Muscle tear, Atypical femoral fracture (bisphosphonate-related) | Covers hip joint to knee joint. To include leg, add a separate request |
| MRI, Thigh, With Contrast | Contrast | Infection, Metastases, Tumor |  |
| MRI, Leg, Routine | Non-contrast | Pain, Muscle tear | Covers knee joint to ankle joint. To include thigh, add a separate request |
| MRI, Leg, With Contrast | Contrast | Infection, Metastases, Tumor |  |
| MRI, Knee, Routine | Non-contrast | Meniscus/ligament tear, Chondromalacia patellae, Fracture, OCD, Pain, Trauma, Baker's cyst | Covers knee joint and its surrounding structures |
| MRI, Knee, With Contrast | Contrast | Bony/soft tissue lesions, PVNS/TSGCT, Inflammation, Infection |  |
| MRI, Ankle, Routine | Non-contrast | Achilles tendinopathy/tear, AVN talus, Ligament/tendon tear, OCD, Plantar fasciitis, Trauma | Covers ankle Joint and its surrounding structures |
| MRI, Ankle, With Contrast | Contrast | Bony/soft tissue lesions, PVNS/TSGCT, Inflammation, Infection |  |
| MRI, Foot, Routine | Non-contrast | - Forefoot: First metatarsophalangeal joint (MTPJ) or lesser MTPJ pathology (plantar plate tears) use the forefoot protocol, Stress fracture of metatarsal - Midfoot: Lisfranc - Hindfoot, midfoot, or forefoot depending on the suspected region: Ligament / tendon tear, Fracture, Pain, Trauma | Specify coverage: Whole foot if unspecified or suspected infection/collection. Hindfoot alone is the same as MRI Ankle |
| MRI, Foot, With Contrast | Contrast | - Forefoot: Morton’s neuroma. - Hindfoot, midfoot, or forefoot depending on the suspected region: Bony and soft tissue lesions, Inflammation (e.g., synovitis, myositis), Infection such as osteomyelitis, cellulitis or septic arthritis. Pigmented villonodular synovitis (PVNS) or tenosynovial giant cell tumor (TSGCT). Plantar fibroma or lesion. |  |
