# Supplementary material for: Initial Insights Into an Institutional Secure Large Language Model for Magnetic Resonance Imaging Examination Requests: Retrospective Study
Source: J Med Internet Res. 2026 Apr 7;28:e82579. doi: 10.2196/82579 (PMC13055936; doi:10.2196/82579)
Supplement: Multimedia Appendix 2 [file jmir-v28-e82579-s002.docx]

**Reason for exam Imaging Reporting and Data System (RI-RADS)**

Each clinician-submitted MER and its sLLM-enriched counterpart were evaluated with the **Reason-for-Exam Imaging Reporting and Data System (RI-RADS)**, a four-level framework developed to classify the information quality of radiology requisitions (Abedi et al., 2019).

**Reference:**

Abedi A, Tofighi S, Salehi S, Latterman PT, Basques KD, Gholamrezanezhad A. Reason for exam Imaging Reporting and Data System (RI-RADS): A grading system to standardize radiology requisitions. Eur J Radiol. 2019;120:108661. doi: 10.1016/j.ejrad.2019.108661.

Abbreviations: MER= MRI examination request; sLLM = secure large language model

**RI-RADS grading system breakdown:**

| **Grade** | **Description** | **Information included in the requisition** | **Examples** |
| --- | --- | --- | --- |
| **A** | Adequate | All key categories of information included | - “Elevated alpha-fetoprotein, history of hepatitis B; query small HCC in segment VIII” (MRI liver with dynamic contrast) - “Three months post-arthroscopic meniscal repair, persistent locking; please assess integrity of repair” (MRI right knee) |
| **B** | Barely adequate | All key categories of information included, some clinical findings missing | - “New-onset diplopia, rule out cavernous-sinus lesion” without duration of symptoms (MRI skull base) - “Left breast invasive ductal carcinoma, staging” but omits prior chemotherapy dates (MRI breast) |
| **C** | Considerably limited | Two categories of information included. | “to rule out vertebral metastasis” for an MRI spine, but does not mention recent spinal instrumentation and radiotherapy. |
| **D** | Deficient | One or no category of information included. | “Left shoulder pain” for an MRI right knee request. History provided is “scan required” or “follow-up” |

Key information categories assessed

1. Impression – working or differential diagnosis
2. Clinical findings
    a. Presenting signs and symptoms
    b. Duration/chronicity of symptoms
    c. Anatomic localisation of symptoms
    d. Relevant past medical or surgical history
    e. Pertinent laboratory data
    f. Prior imaging results (if available)
3. Diagnostic intent – e.g., confirm or exclude a diagnosis, grade or stage a lesion, pre-operative planning, follow-up or treatment response evaluation.

**Examples of Original Clinician versus secure LLM-Augmented MRI Examination Requests (MERs)**

*Representative examples across spine, neuro, and body MRI.*

**Case 1 – Lumbar Spine MRI (prior surgery)**

**Original Clinician MER (RI-RADS D)**

Protocol requested: MRI Lumbar Spine
Special instructions: —
Scheduling instructions: —
Working diagnosis / Reason for exam: L4/5 PID
Relevant clinical information: L4/5 PID
Contrast requested: Not specified
Contraindications to contrast/MRI: None mentioned
Past surgery: *Not mentioned*
Implants / instrumentation: Not mentioned

**LLM-Augmented MER (RI-RADS A)**

Protocol requested: MRI Lumbar Spine, Routine
Special instructions: None
Scheduling instructions: —
Working diagnosis / Reason for exam: Possible L4/5 PID recurrence; chronic low back pain with radicular symptoms
Clinical findings: Ongoing low back pain (VAS 6–7/10); left-dominant posterolateral calf pain
Contrast requested: Non-contrast
Contrast risk: None
Contraindications: None
Past surgery: L2/3, L3/4, L4/5 endoscopic discectomy (2019)
Implants / instrumentation: None
Exam coverage: T12–sacrum

**Discrepancy Statement**

LLM correctly identified prior surgery that was entirely missing from the clinician MER. Contrast not required as surgery performed >5 years previously, but metal artefact reduction sequences can be utilised with a longer slot time planned in advance.

**Case 2 – Pituitary MRI (known adenoma and prior surgery)**

**Original Clinician MER (RI-RADS B)**

Protocol requested: MRI Pituitary
Special instructions: With contrast
Reason for exam: Pituitary adenoma with apoplexy and panhypopituitarism
Relevant clinical information: As above
Contrast requested: Yes
Contraindications: None
Past surgery: *Not mentioned*
Implants / instrumentation: None

**LLM-Augmented MER (RI-RADS B)**

Protocol requested: MRI Pituitary, Routine (Dynamic Contrast)
Reason for exam: Surveillance of known pituitary adenoma
Clinical findings: Known adenoma with prior apoplexy and panhypopituitarism
Contrast requested: Contrast
Contrast risk: None
Contraindications: None
Past surgery: *Not mentioned by LLM (radiologist noted omission)*
Coverage: Pituitary gland and surrounding structures

Implants / instrumentation: None

**Discrepancy Statement**

Both clinician and LLM selected the correct protocol. However, both the clinician and LLM did not capture prior pituitary surgery, which was mentioned in the EMR.

**Case 3 – Uterus/Cervix MRI (Cancer)**

**Original Clinician MER (RI-RADS C)**

Protocol requested: MRI Uterus and Cervix
Special instructions:
Reason for exam: Known grade 3 endometrioid adenocarcinoma
Relevant clinical information: “PET CT with contrast please”
Contrast requested: Not specified
Contraindications: None mentioned
Past surgery: None mentioned
Implants: None

**LLM-Augmented MER (RI-RADS A)**

Protocol requested: MRI Uterus/Cervix – Cancer
Reason for exam: Evaluate grade 3 endometrial cancer
Clinical findings: Thickened endometrium (29 mm); 3.5 × 3.2 × 3.1 cm lesion
Contrast requested: Contrast
Contrast risk: None
Contraindications: None
Coverage: Uterus and cervix

Past surgery: None mentioned
Implants: None

**Discrepancy Statement**

Clinician MER included incorrect and misleading PET-CT statement (under relevant clinical information). LLM corrected this and aligned the request to the appropriate cancer protocol.

**Case 4 – Liver MRI (assessment of suspected liver metatases)**

**Original Clinician MER (RI-RADS B)**

Protocol requested: MRI Liver
Special instructions: With contrast
Reason for exam: Possible relapse of metastatic ascending colon adenocarcinoma
Relevant clinical information: As above
Contrast requested: Yes
Contraindications: None mentioned

**LLM-Augmented MER (RI-RADS A)**

Protocol requested: MRI Liver, Routine
Reason for exam: Evaluate FDG-avid segment III lesion on PET-CT
Clinical findings: Subcapsular segment III lesion; possible recurrence of colon cancer
Contrast requested: Contrast
Contrast risk: None
Coverage: Liver

Contraindications: None mentioned

**Discrepancy Statement**

Clinician MER omitted known PET-CT findings. Secure LLM added them, improving clarity for the specific MRI request and reporting radiologist.

**Case 5 – Cervical Cancer (Renal Impairment)**

**Original Clinician MER (RI-RADS C)**

Protocol requested: MRI Pelvis (non-contrast)
Reason for exam: Recurrent cervical cancer
Relevant clinical information: PET-CT shows FDG-avid cervical lesions
Contrast requested: Non-contrast
Contraindications: *Not stated (renal failure omitted)*

**LLM-Augmented MER (RI-RADS A)**

Protocol requested: MRI Uterus/Cervix – Cancer
Reason for exam: Evaluate FDG-avid cervical lesions; biopsy planning
Clinical findings: Recurrent cervical cancer; PET-CT findings
Contrast requested: Non-contrast due to renal impairment (eGFR 31–48)
Contrast risk: Renal impairment documented
Coverage: Pelvis focused on cervix

**Discrepancy Statement**

Clinician MER incorrectly ordered a general pelvis MRI and did not mention renal impairment as the reason for non-contrast. LLM selected the correct cancer protocol and correctly suggested a non-contrast study due to renal failure (institutional protocol).

**Case 6 – Crohn’s Disease (Perianal Disease)**

**Original Clinician MER (RI-RADS C)**

Protocol requested: MRI Pelvis
Reason for exam: Crohn disease with recent flare
Relevant clinical info: Perianal skin tag, possible discharge
Contrast requested: Unspecified
Contraindications: None mentioned

**LLM-Augmented MER (RI-RADS A)**

**Protocol requested:**

- MRI Rectum & Perineum – Fistula protocol
- MRI Enterography, Routine
  Reason for exam: Evaluate perianal disease/fistula + Crohn’s flare
  Clinical findings: Loose stool; perianal skin tag; possible discharge
  Contrast requested: Contrast (both exams)
  Contrast risk: None
  Coverage: Rectum, perineum, small bowel

**Discrepancy Statement**

Clinician MER ordered MRI Pelvis, which is inappropriate. LLM correctly selected two required protocols (identified perianal fistula in the notes) for comprehensive Crohn’s assessment.

**Case 7 – Cervical Spine MRI (Instrumentation)**

**Original Clinician MER (RI-RADS C)**

Protocol requested: MRI Cervical Spine
Reason for exam: Neck pain, progressive neurology
Relevant clinical info: As above
Contrast requested: Unspecified
Contraindications: None
Past surgery: *Not mentioned* (ACDF C5–C7 present)

**LLM-Augmented MER (RI-RADS A)**

Protocol requested: MRI Cervical Spine, Routine
Reason for exam: Suspected cervical spondylosis and radiculopathy
Clinical findings: Neck pain; neck stiffness; numbness R forearm; thumb/index finger weakness
Contrast requested: Non-contrast
Contrast risk: None
Contraindications: None
Past surgery: Spinal instrumentation at C5–C7 (correctly detected)

**Discrepancy Statement**

Clinician MER did not mention prior ACDF. LLM identified the instrumentation, improving protocol safety and completeness. This will also allow metal artefact reduction to be planned into the protocol.
